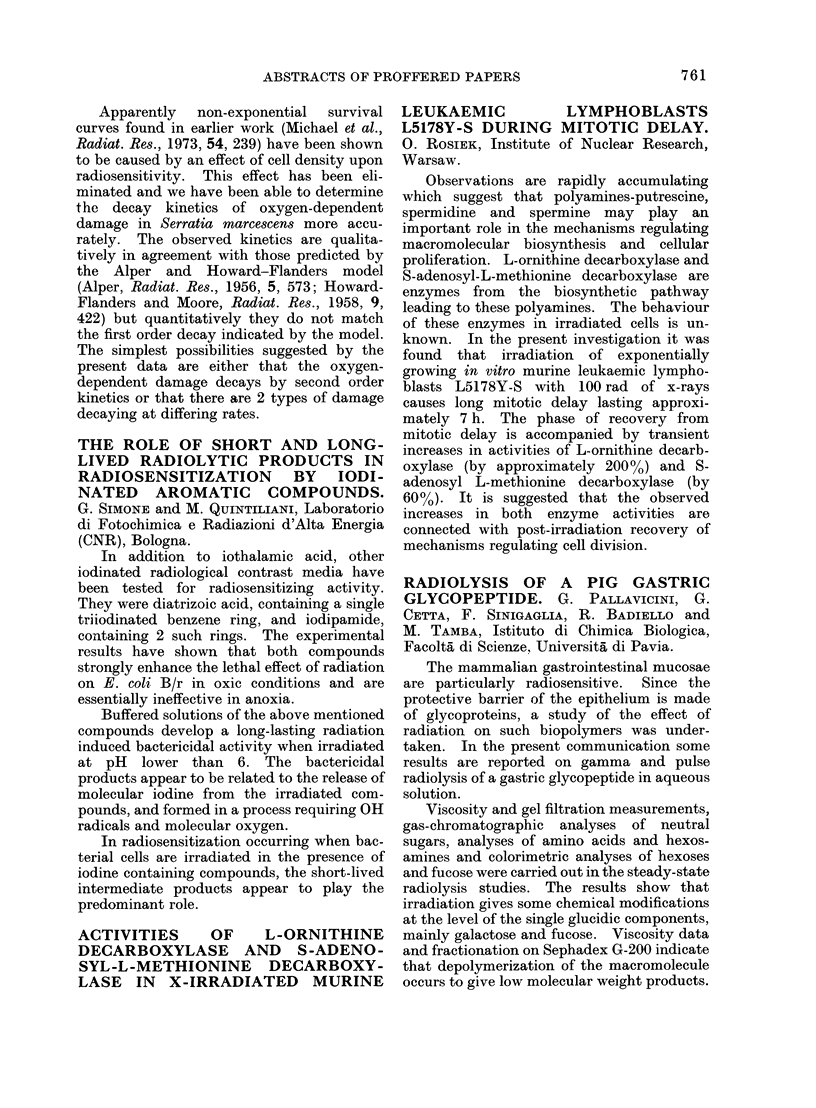# Proceedings: The role of short and long-lived radiolytic products in radiosensitization by iodinated aromatic compounds.

**DOI:** 10.1038/bjc.1975.322

**Published:** 1975-12

**Authors:** G. Simone, M. Quintilliani


**THE ROLE OF SHORT AND LONG-LIVED RADIOLYTIC PRODUCTS IN RADIOSENSITIZATION BY IODINATED AROMATIC COMPOUNDS.** G. SIMONE and M. QUINTILIANI, Laboratorio di Fotochimica e Radiazioni d'Alta Energia (CNR), Bologna.

In addition to iothalamic acid, other iodinated radiological contrast media have been tested for radiosensitizing activity. They were diatrizoic acid, containing a single triiodinated benzene ring, and iodipamide, containing 2 such rings. The experimental results have shown that both compounds strongly enhance the lethal effect of radiation on *E. coli* B/r in oxic conditions and are essentially ineffective in anoxia.

Buffered solutions of the above mentioned compounds develop a long-lasting radiation induced bactericidal activity when irradiated at pH lower than 6. The bactericidal products appear to be related to the release of molecular iodine from the irradiated compounds, and formed in a process requiring OH radicals and molecular oxygen.

In radiosensitization occurring when bacterial cells are irradiated in the presence of iodine containing compounds, the short-lived intermediate products appear to play the predominant role.